# Risk factors for hip fractures: the role of femoral and acetabular morphology in predicting proximal femur fracture types

**DOI:** 10.1177/11207000251389829

**Published:** 2025-11-12

**Authors:** Nele Wagener, Vincent Leopold, Felix Rarreck, Torsten Diekhoff, Sebastian Hardt

**Affiliations:** 1 Department of Orthopaedic Surgery, Centre for Musculoskeletal Surgery, Charité - Universitätsmedizin Berlin, Berlin, Germany; 2 Department of Radiology, Charité - Universitätsmedizin Berlin, Berlin, Germany

**Keywords:** Developmental dysplasia of the hip (DDH), femoral and acetabular morphology, fracture localisation, hip biomechanics, proximal femur fractures, radiographic parameter

## Abstract

**Introduction:**

Due to demographic shifts and an increasing proportion of older adults at higher fracture risk, the overall burden of proximal femur fractures has risen significantly in recent years, placing a burden on patients and healthcare systems. Despite extensive research on hip fracture epidemiology and treatment, the specific influences of femoral and acetabular parameters on fracture localisation remain underexplored. This study evaluates the impact of these parameters on proximal femur fracture localisation.

**Materials and methods:**

This retrospective cohort study analysed data from 400 patients with proximal femur fractures, including medial and lateral femoral neck fractures (FNFs), pertrochanteric, and subtrochanteric fractures, treated at a university hospital between 2010 and 2022. Radiographic measurements of femoral head size and other morphological parameters were conducted using pre-operative pelvic radiographs. Statistical analyses included multivariate logistic regression.

**Results:**

A larger head-radius vertical (HRV) was associated with an increased risk of lateral FNFs (OR 1.11; *p* = 0.007). A lower Femoral Head Protrusion Index (FHEI) significantly increased the risk of lateral (OR 0.87; *p* < 0.001), pertrochanteric (OR 0.88; *p* < 0.001), and subtrochanteric fractures (OR 0.86; *p* < 0.001). Femur canal width was associated with a higher risk of pertrochanteric fractures (OR 1.17; *p* = 0.001), and a smaller lateral centre edge angle (LCEA) correlated with higher risks of lateral (OR 0.92; *p* = 0.021) and pertrochanteric fractures (OR 0.92; *p* = 0.018). Additionally, a higher body mass index (BMI) was significantly associated with subtrochanteric fractures (or 1.07; *p* = 0.020).

**Conclusions:**

The study identified key variables associated with proximal femur fracture localisation. Morphological parameters such as HRV, FHEI, canal width, and LCEA, as well as BMI for subtrochanteric fractures, significantly influence fracture localisation, highlighting their importance for personalised risk assessment and preventive strategies.

## Introduction

The localisation of proximal femur fractures varies significantly, influenced by numerous anatomical and biomechanical factors.^1^ While much research addresses hip fracture epidemiology and treatment, the impact of specific femoral and acetabular parameters on fracture localisation remains underexplored.

Developmental dysplasia of the hip (DDH), characterised by insufficient femoral head coverage, can significantly alter hip biomechanics, increasing susceptibility to specific fracture sites.^2–4^ Additionally, osteoporosis and hip osteoarthritis are key contributors to proximal femur fractures. Osteoporosis, marked by reduced bone density, substantially increases fracture risk,^5^ while osteoarthritis affects joint load and wear.^6^ Parameters like femoral shaft angle and acetabular coverage are essential for understanding fracture mechanisms.

Globally, an estimated 1.6 million hip fractures occur annually, a figure expected to rise due to aging populations and increasing osteoporosis prevalence.^7,8^ In the United States, hip fractures rank among the top 20 most costly medical conditions, with annual costs nearing 20 billion dollars.^9–11^ By 2030, annual hip fracture cases in the USA are projected to reach 300,000, underscoring the need for improved prevention and management strategies.^12^

This study investigates how femoral and acetabular parameters, along with DDH, osteoporosis, and hip osteoarthritis, influence fracture localisation. Through clinical data and conventional diagnostic methods such as radiographs, we aim to clarify the biomechanical relationships and hypothesise that these factors have a significant impact on fracture localisation, thus aiding in the development of tailored prevention and treatment strategies.

## Methods

**Figure 1.**
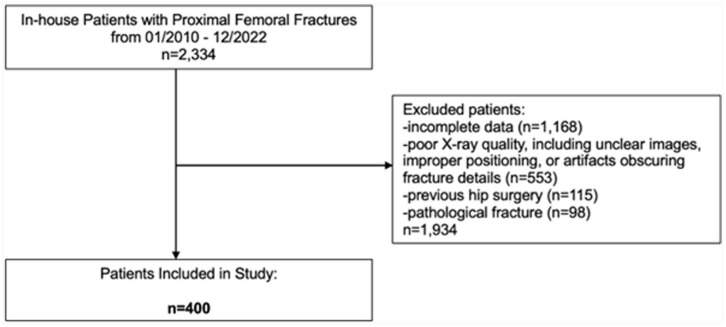
Flowchart of patient selection process.

This retrospective study included 400 patients (225 females, 175 males; mean age 74.44 ± 12.34 years) with medial, lateral, pertrochanteric, or subtrochanteric femur fractures treated surgically from 2010–2022. Cases with incomplete data, poor radiograph quality, prior hip surgeries, or pathological fractures were excluded. Patients were evenly grouped by fracture type. Details are in Table 1 and Figure 1.

**Table 1.** Health condition profiles across different proximal femur fracture type.

Characteristic		Type of proximal femur fracture				*p-value*
		Medial	Lateral	Pertrochanteric	Subtrochanteric	
		*n = 100*	*n = 100*	*n= 100*	*n = 100*	
Localisation	L	46 (46.00%)	39 (39.00%)	53 (53.00%)	51 (51.00%)	0.179
	R	54 (54.00%)	61 (61.00%)	46 (46.00%)	49 (49.00%)	
Sex	m	44 (44.00%)	50 (50.00%)	38 (38.00%)	43 (43.00%)	0.419
	f	56 (56.00%)	50 (50.00%)	62 (62.00%)	57 (57.00%)	
Osteoarthritis (Kellgren & Lawrence)	0	19 (19.00%)	14 (14.00%)	5 (5.00%)	7 (7.00%)	0.028
	1	27 (27.00%)	25 (25.00%)	44 (44.00%)	30 (30.00%)	
	2	44 (44.00%)	51 (51.00%)	41 (41.00%)	46 (46.00%)	
	3	9 (9.00%)	9 (9.00%)	8 (8.00%)	15 (15.00%)	
	4	1 (1.00%)	1 (1.00%)	2 (2.00%)	2 (2.00%)	
THA on healthy side	Yes/No	4/95 (4.04%)	3/91 (3.19%)	3/95 (3.06%)	3/92 (3.16%)	>0.999
Cardiovascular disease	Yes/No	58/42 (58.00%)	45/55 (45.00%)	51/49 (51.00%)	47/53 (47.00%)	0.274
Respiratory disease	Yes/No	16/84 (16.00%)	23/77 (23.00%)	15/85 (15.00%)	10/90 (10.00%)	0.101
Digestive disease	Yes/No	3/97 (3.00%)	9/91 (9.00%)	6/94 (6.00)	7/93 (7.00%)	0.402
Neurological disease	Yes/No	22/78 (22.00%)	15/85 (15.00%)	23/77 (23.00%)	15/85 (15.00%)	0.304
Endocrine disorder	Yes/No	29/71 (29.00%)	17/83 (17.00%)	20/80 (20.00%)	23/77 (23.00%)	0.214
Musculoskeletal disorder	Yes/No	21/79 (21.00%)	28/72 (28.00%)	20/80 (20.00%)	17/83 (17.00%)	0.293
Psychiatric disorders	Yes/No	5/95 (5.00%)	6/94 (6.00%)	4/96 (4.00%)	7/93 (7.00%)	0.882
Kidney diseases	Yes/No	16/84 (16.00%)	24/76 (24.00%)	23/77 (23.00%)	13/87 (13.00%)	0.143
Infectious diseases	Yes/No	11/89 (11.00%)	5/95 (5.00%)	7/93 (7.00%)	2/98 (2.00%)	0.071
Oncological diseases	Yes/No	6/94 (6.00%)	10/90 (10.00%)	8/92 (8.00%)	4/96 (4.00%)	0.402
Osteoporosis	Yes/No	10/90 (10.00%)	13/87 (13.00%)	9/91 (9.00%)	9/91 (9.00%)	0.780
Smoking	Yes/No	25/75 (25.00%)	23/76 (23.23%)	21/79 (21.00%)	13/87 (13.00%)	0.161

THA, total hip arthroplasty.

Note: The table highlights correlations between health conditions and fracture types. Distribution of health conditions across proximal femur fracture types (medial, lateral, pertrochanteric, subtrochanteric) is shown, using chi-square tests to assess significance (*p* < 0.05).

**Figure 2.**
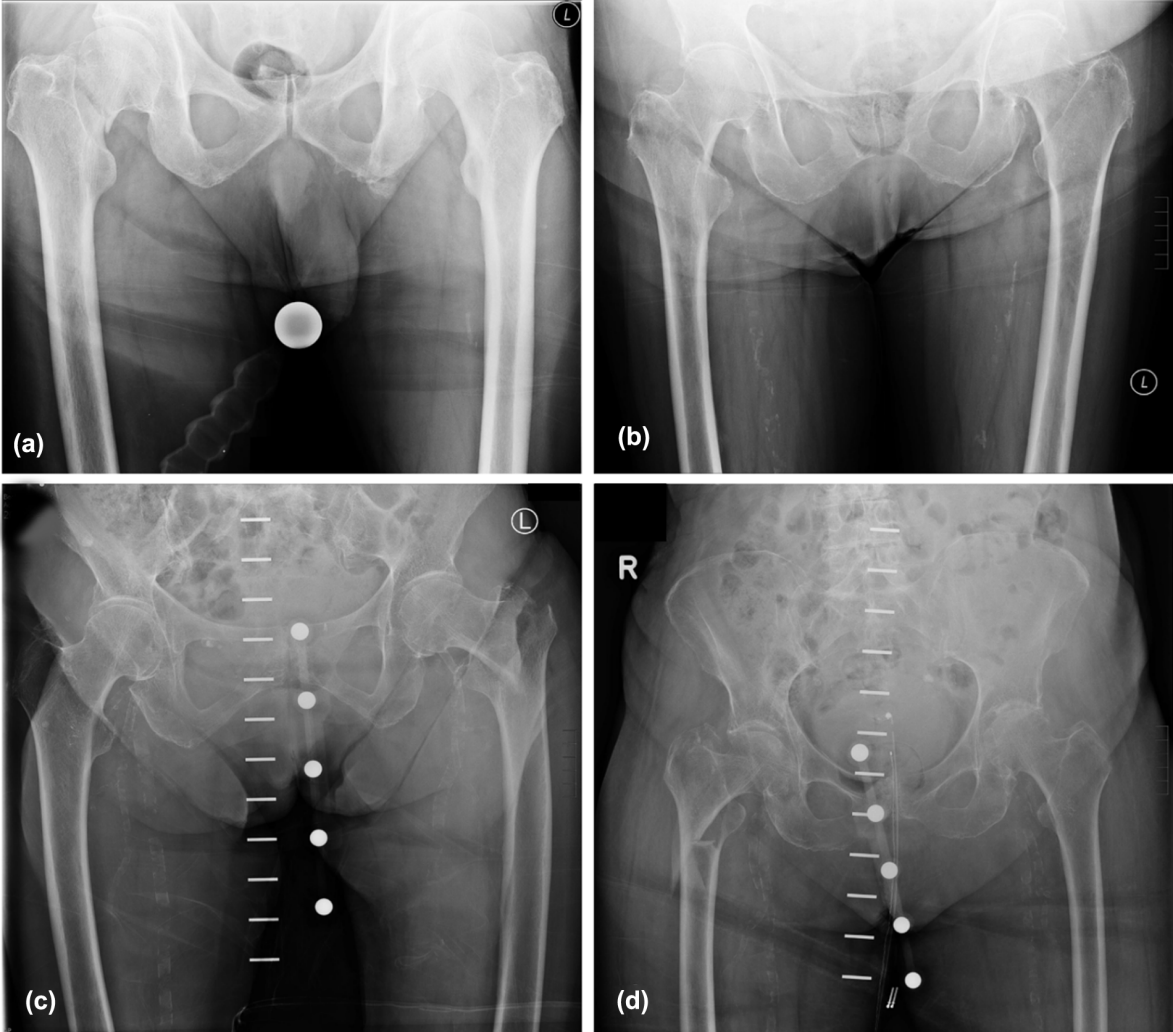
Radiographic images showing different types of proximal femur fractures. (a) Medial femoral neck fracture, (b) Lateral femoral neck fracture, (c) Pertrochanteric femoral fracture, and (d) Subtrochanteric femoral fracture.

Preoperative standardised anteroposterior pelvic radiographs (100 cm distance, hips internally rotated 15–30°) were analysed using MERLIN (Phönix-PACS GmbH). Femoral and acetabular parameters were measured independently by 2 orthopaedic specialists, with averages calculated. The obturator foramen index was applied for standardisation (Figure 2).

**Figure 3.**
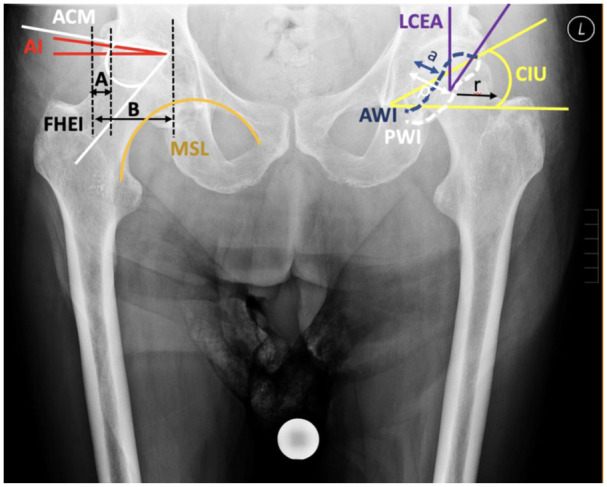
The figure highlights key parameters for hip joint assessment: LCEA (lateral centre-edge angle), AI (acetabular index), CIU (cup inclination angle by Ullmann), ACM (acetabular roof angle by Idelberger and Frank), FHEI (femoral head extrusion index), AWI (anterior wall index), PWI (posterior wall index), Menard-Shenton Line, and the Cross-over Sign for acetabular retroversion. These metrics are crucial for diagnosing and evaluating hip dysplasia, osteoarthritis, and related conditions.

**Figure 4.**
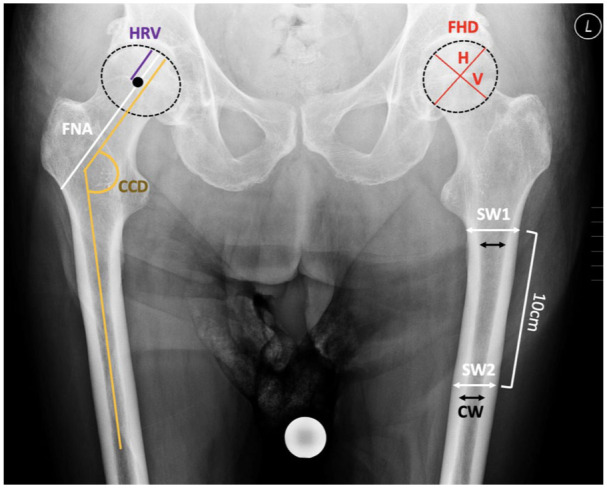
The morphology of the proximal femur was analysed using the following parameters: femoral head diameter (FHD) in vertical (V) and horizontal (H) dimensions (mm), femoral neck axis length (FNA) (mm), head radius vertical (HRV) (mm), CCD angle (°), HRV/FNA ratio, shaft width at the lower edge of the lesser trochanter (SW1), shaft width 10 cm distal to the lesser trochanter (SW2), and canal width (CW).

To analyse the proximal femur and acetabulum morphology, the following parameters were assessed: femoral head diameter (FHD), divided into vertical (V) and horizontal (H);^13,14^ femoral neck axis length (FNA);^15^ head radius vertical (HRV);^16^ caput-collum-diaphyseal angle (CCD);^17^ HRV/FNA; shaft width at the lesser trochanter (SW1) and 10 cm distally (SW2); and canal width (CW).^18^ Acetabular parameters included the lateral centre-edge angle (LCEA),^19^ acetabular index (AI),^19^ cup inclination angle (CIU),^20^ acetabular roof angle (ACM),^21^ femoral head extrusion index (FHEI) as (A/B) x 100,^22^ anterior wall index (AWI) as a/r, and posterior wall index (PWI) as p/r.^23^ Hip alignment was evaluated using the Menard-Shenton line,^24^ measured on both fractured and healthy sides, and the crossover sign,^25^ indicating acetabular retroversion (Figure 3). Femoral bone quality was classified into Dorr types A, B, and C, based on the ratio of the shaft width 10 cm below the lesser trochanter to the canal width (Figure 4).^26^ Patient comorbidities were also recorded. The study was approved by the local ethics committee (EA1/203/23), with informed consent waived due to its retrospective nature. All procedures adhered to the Declaration of Helsinki.

### Statistical methods

Categorical data were summarised as absolute (*n*) and relative frequencies (%), and continuous variables as mean (M) ± standard deviation (SD). Normality was assessed using the Shapiro-Wilk test, skewness, kurtosis, and histograms. Chi-Square or Fisher’s exact test (for frequencies <5) compared categorical variables, while 1-way ANOVA with Bonferroni-adjusted post-hoc tests analysed continuous outcomes. Effect sizes (η²) were classified as small (⩾0.01), medium (⩾0.06), or large (⩾0.14). Spearman correlations (95% confidence interval [CI]) assessed relationships between metric variables. Statistical significance was set at *p* < 0.05. Analyses were performed using SPSS v29 (IBM).

## Results

### Patient-specific prevalence of health conditions across different types of proximal femur fractures

No significant differences were observed in localisation (*p* = 0.179), sex distribution (*p* = 0.419), THA (*p* > 0.999), or comorbidities across fracture types. Only osteoarthritis significantly correlated with fracture type (Table 2).

**Table 2:** Demographic characteristics: age and BMI variation among proximal femur fracture types.

	medial	lateral	pertrochantaric	subtrochantaric	*p*-value	Lateral vs. pertrochanteric	pertrochanteric vs. subtrochanteric
	mean ± SD	mean ± SD	mean ± SD	mean ± SD			
Age (years)	75.07 ± 23.99	71.05 ± 24.35	79.52 ± 23.52	72.15 ± 25.40	**<0.001**	**<0.001**	**0.002**
BMI (kg/m2)	23.99 ± 4.14	24.44 ± 4.65	23.56 ± 4.33	25.40 ± 5.02	0.072	0.221	**0.015**

SD, standard deviation; BMI, body mass index.

Note: The table shows the mean and SD of age and BMI for medial, lateral, pertrochanteric, and subtrochanteric fractures. From ANOVA and post hoc pairwise comparisons, significant differences between fracture types are highlighted in bold (p <0.05).

### Evaluation of acetabular radiographic markers in different types of hip fractures

No significant differences were found for posterior wall sign (*p* = 0.284), crossover sign (*p* = 0.262), Shenton line disruption (*p* = 0.481), or protrusio acetabuli (*p* = 0.121) across fracture types (Table 3).

**Table 3:** Radiological markers and their distribution in proximal femur fracture subtypes.

Radiological marker		medial	lateral	pertrochanteric	subtrochanteric	*p-value*
		*n (%)*	*n (%)*	*n (%)*	*n (%)*	
Posterior wall sign (Fracture side)	negative	74 (74.00%)	79 (79.00%)	67 (67.00%)	71 (71.00%)	0.284
	positive	26 (26.00%)	21 (21.00%)	33 (33.00%)	29 (29.00%)	
Crossover sign (fracture side)	negative	86 (86.00%)	84 (84.00%)	83 (83.00%)	76 (76.00%)	0.262
	positive	14 (14.00%)	16 (16.00%)	17 (17.00%)	24 (24.00%)	
Shenton line (fracture side)	interrupted	59 (59.00%)	22 (22.00%)	19 (19.00%)	17 (17.00%)	**<0.001**
	normal	41 (41.00%)	78 (78.00%)	81 (81.00%)	83 (83.00%)	
Shenton line (healthy side)	interrupted	1 (1.00%)	2 (2.00%)	2 (2.00%)	5 (5.00%)	0.481
	normal	99 (99.00%)	98 (98.00%)	98 (98.00%)	94 (94.00%)	
Protrusio acetabuli (fracture side)	negative	100 (100.00%)	97 (97.00%	97 (97.00%)	94 (94.00%)	0.121
	positive	0 (0.00%)	3 (3.00%)	3 (3.00%)	6 (6.00%)	

Analysis of radiographic signs across fracture types, showing frequencies (*n*, %) of positive and negative observations for posterior wall sign, cross-over sign, Shenton line (fracture and healthy sides), and protrusio acetabuli.

Value in bold indicates statistical significance.

### Significant differences in acetabular angles and indices across hip fracture types

LCEA differed significantly (*p* = 0.007), with subtrochanteric fractures having the highest mean. CIU showed significant differences (*p* = 0.028), particularly between pertrochanteric and medial, and subtrochanteric and medial fractures. ACM was significantly lower in subtrochanteric compared to lateral fractures (*p* = 0.026). FHEI exhibited highly significant differences (*p* < 0.001), with medial fractures differing from lateral, pertrochanteric, and subtrochanteric fractures. PWI also differed significantly (*p* = 0.027), especially between subtrochanteric and medial, and subtrochanteric and lateral fractures (Table 4).

**Table 4:** Acetabular morphological parameters across proximal femur fracture.

Acetabular parameter (Fracture side)	medial	lateral	pertrochanteric	subtrochanteric	*p*-value
	mean ± SD	mean ± SD	mean ± SD	mean ± SD	
LCEA (Fracture side)	34.58 ± 6.95	35.46 ± 6.80	35.82 ± 6.96	38.00 ± 8.01	**0.007**
AI (Fracture side)	5.46 ± 4.90	5.19 ± 4.38	5.98 ± 4.41	4.61 ± 5.21	0.227
CIU(Fracture side)	52.95 ± 4.74	53.47 ± 3.40	54.67 ± 4.11	54.20 ± 5.00	0.028
ACM (Fracture side)	45.75 ± 4.31	45.12 ± 3.04	45.83 ± 3.57	44.45 ± 3.53	0.026
FHEI(Fracture side)	15.14 ± 9.81	11.36 ± 6,90	9.96 ± 6.28	9.04 ± 7.00	**<0.001**
AWI (Fracture side)	15.66 ± 5.78	15.70 ± 5.81	16.72 ± 6.05	16.64 ± 5.46	0.394
PWI (Fracture side)	24.97 ± 4.93	24.61 ± 5.64	26.73 ± 6.07	26.07 ± 5.58	**0.027**

SD, standard deviation; LCEA, lateral centre-edge angle; AI, acetabular index; CIU, cup inclination angle; FHEI, femoral head protrusion index; AWI, anterior wall index; PWI, posterior wall index.

Note: Comparative analysis of proximal femur anatomy across fracture types (medial, lateral, pertrochanteric, subtrochanteric). Values in bold indicate statistical significance.

### Distribution of canal shape by Dorr classification

The majority of fractures were classified as Dorr B: medial (71.72%), lateral (75.76%), pertrochanteric (76.77%), and subtrochanteric (79.00%). A trend toward significance in canal shape distribution was observed (*p* = 0.060), but it was not statistically significant (Table 5).

**Table 5.** Canal morphology based on Dorr classification in relation to proximal femur fracture types.

Canal shape (Dorr classification)		medial	lateral	pertrochantaric	subtrochanteric	
		*n*	*n*	*n*	*n*	*p*-value
Canal shape according to Dorr et al.	A	20 (20.20%)	10 (10.10%)	7 (7.07%)	9 (9.00%)	0.060
	B	71 (71.72%)	75 (75.76%)	76 (76.77%)	79 (79.00%)	
	C	8 (8.08%)	14 (14.14%)	16 (16.16%)	12 (12.00%)	

Distribution of canal shapes (A, B, and C) according to the Dorr classification for medial, lateral, pertrochanteric, and subtrochanteric fractures. Most fractures were classified as Dorr B.

### Detailed comparison of femoral anatomical parameters across various fracture types

Lateral fractures had the largest femoral head diameter (FHD horizontal, *p* = 0.028). Subtrochanteric fractures showed the smallest head radius vertical (HRV, *p* < 0.001). Canal width (CW) was largest in lateral fractures (*p* = 0.001). These findings emphasise the role of morphometric analysis in fracture assessment (Table 6).

**Table 6.** Detailed comparative analysis of femoral anatomical parameters by fracture type.

Femoral anatomical parameter	medial	lateral	pertrochantaric	subtrochantaric	p-value
	mean ± SD	mean ± SD	mean ± SD	mean ± SD	
CCD (°) (healthy side)	133.65 ± 5.59	133.31 ± 5.80	133.27 ± 6.08	132.72 ± 6.33	**0.740**
FNA (mm) (healthy side)	112.57 ± 11.29	113.38 ± 12.82	111.04 ± 11.40	110.6 ± 10.54	**0.287**
FHD horizontal (healthy side)	55.11 ± 4.98	55.59 ± 5.75	54.41 ± 6.50	53.33 ± 5.07	**0.028**
HRV (fracture side)	27.55 ± 2.50	27.8 ± 2.87	27.21 ± 3.25	26.66 ± 2.54	0.028
HRV/FNA (fracture side)	0.25 ± 0.02	0.25 ± 0.02	0.25 ± 0.03	0.24 ± 0.02	0.384
FHD horizontal (fracture side)	54.68 ± 5.44	55.23 ± 6.35	53.93 ± 4.87	58.18 ± 52.63	0.693
FHD vertical (fracture side)	46.08 ± 6.28	48.51 ± 6.14	44.91 ± 4,81	47.46 ± 5.14	**<0.001**
SW1 (fracture side)	36.11 ± 3.97	36.53 ± 3.68	36.58 ± 3.93	36.57 ± 3.64	0.792
CI (fracture side)	0.42 ± 0.22	0.43 ± 0.12	0.38 ± 0.11	0.42 ± 0.10	0.149
SW2 (fracture side)	30.35 ± 4.25	31.22 ± 3.70	31.42 ± 31.42	31.73 ± 3.63	0.058
CW (fracture side)	17.25 ± 3.48	17.88 ± 3.79	19.42 ± 4.09	18.35 ± 3.83	**0.001**

CCD, caput-collum-diaphyseal angle; FNA, femoral neck axis length; HRV, head radius vertical; FHD, femoral head diameter; SW, shaft width; CI, cortical index; CW, canal width.

Note: Values in bold indicate statistical significance.

### Multinomial regression with median as reference category

The multinomial regression analysis identified significant predictors for specific types of proximal femur fractures. Lateral fractures were associated with a larger head-radius vertical (HRV) (OR 1.11; *p* = 0.007), smaller lateral centre-edge angle (LCEA) (OR 0.92; *p* = 0.021), and lower femoral head extrusion index (FHEI) (OR 0.87; *p* < 0.001). Pertrochanteric fractures were significantly linked to larger canal width (OR 1.17; *p* = 0.001), smaller LCEA (OR 0.92; *p* = 0.018), and lower FHEI (OR 0.88; *p* < 0.001). For subtrochanteric fractures, a higher BMI (OR 1.07; *p* = 0.020) and a lower FHEI (OR 0.86; *p* < .001) were significantly associated. In contrast to previous assumptions, BMI was not significantly associated with lateral (OR 1.01; *p* = .819) or pertrochanteric fractures (OR 0.95; *p* = .077). These results highlight HRV, LCEA, and FHEI as key anatomical parameters for lateral fractures; CW, LCEA, and FHEI for pertrochanteric fractures; and BMI and FHEI for subtrochanteric fractures as significant factors influencing fracture localisation (Table 7).

**Table 7 .** Multivariate regression analysis identifying risk factors for proximal femur fracture types

Predictor		OR (95% confidence interval)	p-value
Lateral	Age (years)	0.98 (0.96–1.01)	0.131
	BMI (kg/m2)	1.01 (0.95–1.06)	0.819
	HRV (fracture side)	1.11 (1.03–1.20)	**0.007**
	CW (fracture side)	1.00 (0.91–1.11)	0.944
	LCEA (fracture side)	0.92 (0.87–0.99)	**0.021**
	FHEI (fracture side)	0.87 (0.81–0.93)	**<0.001**
	No osteoporosis vs. osteoporosis	0.55 (0.19–1.57)	0.260
	Men vs. women	0.93 (0.40–2.20)	0.872
Pertrochanteric	Age (years)	1.02 (0.99–1.04)	0.230
	BMI (kg/m2)	0.95 (0.89–1.01)	0.077
	HRV (fracture side)	0.93 (0.86–1.01)	0.071
	CW (fracture side)	1.17 (1.06–1.29)	**0.001**
	LCEA (fracture side)	0.92 (0.86–0.99)	**0.018**
	FHEI (fracture side)	0.88 (0.82–0.94)	**<0.001**
	No osteoporosis vs. osteoporosis	2.11 (0.67–6.66)	0.202
	Men vs. women	1.81 (0.75–4.36)	0.186
Subtrochanteric	Age (years)	0.99 (0.96–1.01)	0.306
	BMI (kg/m2)	1.07 (1.01–1.13)	**0.020**
	HRV (fracture side)	1.07 (0.99–1.16)	0.085
	CW (fracture side)	1.03 (0.93–1.13)	0.618
	LCEA (fracture side)	0.95 (0.89–1.01)	0.096
	FHEI (fracture side)	0.86 (0.80–0.92)	**<0.001**
	No osteoporosis vs. osteoporosis	1.69 (0.48–5.89)	0.414
	Men vs. women	0.94 (0.40–2.23)	0.891

OR, odds ratio; BMI, body mass index; HRV, head radius vertical; CW, canal width; LCEA, lateral centre-edge angle; FHEI, femoral head protrusion index.

Note: This table summarises multivariate analysis results identifying risk factors for lateral, pertrochanteric, and subtrochanteric femoral fractures compared to medial fractures. Values in bold indicate statistical significance.

## Discussion

The study identified key variables associated with proximal femur fracture localisation. A higher HRV measurement significantly increased the risk of lateral fractures, while a lower FHEI was associated with elevated risks for lateral, pertrochanteric, and subtrochanteric fractures. Additionally, a higher CW was linked to a greater risk of pertrochanteric fractures, and a smaller LCEA correlated with increased risks for lateral and pertrochanteric fractures. Furthermore, a higher BMI was significantly associated with an increased risk of subtrochanteric fractures, whereas no significant association with BMI was observed for lateral or pertrochanteric fractures. Regarding acetabular parameters, the CIU angle was significantly higher in pertrochanteric fractures compared to medial fractures, while the ACM was lower in subtrochanteric fractures relative to lateral fractures.

Age differences were also significant, with lateral and pertrochanteric fractures differing markedly, as well as pertrochanteric and subtrochanteric fractures, highlighting age as a potential influence on fracture type. These findings underscore the importance of femoral and acetabular morphological parameters, such as LCEA, FHEI, and ACM, in predicting fracture risk and localisation.

A pathologically low FHEI reflects excessive femoral head protrusion into the acetabulum,^27,28^ disrupting hip joint biomechanics and increasing fracture risk, particularly in the lateral and pertrochanteric regions. This condition alters force distribution, elevating stress on the acetabulum, which can lead to microfractures and, ultimately, larger fractures.

Structural abnormalities like dysplasia and degenerative conditions such as osteoarthritis further exacerbate these risks. Osteoarthritis alters the femoral head’s shape and acetabular structure,^29^ compounding biomechanical instability. In older patients or those with osteoporosis, reduced bone density coupled with abnormal FHEI magnifies fracture susceptibility, as osteoporotic bones are less resistant to stress from minor impacts.

The importance of the FHEI has been highlighted by previous studies. Laborie et al.^29^ found that an increased FHEI is associated with hip joint instability.^30,31^ The clinical significance of a pathological FHEI is that it indicates excessive joint translation and instability in the hip joint, which can lead to abnormal cartilage loading and ultimately osteoarthritis. In the study by Zhang et al.^31^, it is reported that a higher BMI is associated with an increased FHEI, indicating higher degree of dysplasia and instability of the hip, which is further exacerbated by axial loading.

The LCEA is an essential radiological parameter that describes the coverage of the femoral head by the acetabulum.^32–34^ In the present study, it was found that a smaller LCEA is significantly associated with a higher risk of lateral and pertrochanteric hip fractures.

A pathologically reduced LCEA indicates insufficient coverage of the femoral head by the acetabulum. This means that the femoral head receives less stability and support from the acetabulum, which significantly affects the biomechanics of the hip joint.^35^ Clinically, a reduced LCEA can lead to increased load and stress on the acetabulum and femoral head, thereby increasing the risk of osteoarthritis due to uneven distribution of mechanical loads.^33^ These biomechanical changes also heighten the risk of lateral and pertrochanteric fractures because the hip joint is less able to distribute mechanical stresses evenly. Our findings indicate that a lower LCEA correlates with a higher incidence of lateral and pertrochanteric fractures, underscoring the importance of adequate acetabular coverage in preventing such injuries. Patients with a pathologically reduced LCEA may therefore be more susceptible to fractures from minor traumas; however, bone quality also plays a significant role.

The clinical significance of the LCEA is supported by several previous studies. Tannast et al.^28^ found that a lower LCEA is characteristic of an increased risk of hip dysplasia.^35,36^ Maeyama et al.^34^ also demonstrated that the LCEA is a crucial indicator of hip joint instability.

In our study, it was found that the HRV is a significant predictor of certain proximal femur fractures. Specifically, an increased HRV was significantly associated with a higher risk of lateral femur fractures (OR 1.11; *p* = 0.007), whereas no significant association was found for other fracture types.

An increased vertical FHD indicates a larger femoral head, which alters force distribution in the hip joint and elevates the risk of lateral fractures due to higher stress on the lateral femur. A pathologically small femoral head could theoretically compromise joint stability and increase the risk of other fracture types, although our study did not identify a significant association.

Previous studies have also highlighted the importance of the FHD vertical in predicting hip fractures. For instance, a study by Cukurlu et al.^37^ found that an enlarged femoral head is a significant risk factor for hip fractures, particularly in elderly patients with osteoporosis.

In our study, a larger femoral CW was significantly associated with an increased risk of pertrochanteric fractures (OR 1.17; *p* = 0.001). Conversely, subtrochanteric fractures did not show a significant association with canal width. A pathologically reduced CW, described as Type C in the Dorr classification, is typically associated with thinner cortical bone and osteopenia, which increase bone fragility.^38^ Patients with such characteristics are more susceptible to fractures from minor trauma. Therefore, preoperative assessment of canal width can help identify high-risk patients and enable preventive measures to be taken.

Unlike our study, Patel et al.^39^ did not find significant correlations of femoral head diameter (FHD), femoral neck shaft (FNS), and neck-shaft angle (NSA) with any type of hip fracture. Our findings underscore the importance of detailed morphometric analysis in predicting the risk and localisation of various types of hip fractures. The discrepancies between our results and those of Patel et al.^39^ could be attributed to differences in study populations, measurement techniques, or sample sizes. These differences highlight the need for further research to validate the predictive value of these parameters across diverse populations.^39^

The results of our study and those of Tokyay et al.^40^ indicate that certain morphological parameters of the femur and acetabulum are significantly associated with the risk of various types of hip fractures. Our study identified that a larger HRV and a lower FHEI increase the risk for lateral, pertrochanteric, and subtrochanteric fractures. Tokyay et al.^40^ found that a higher AI is associated with an increased risk for trochanteric fractures. A significant difference lies in the parameters examined: while our study measures HRV and FHEI in detail, Tokyay et al.^40^ focus on the neck-shaft angle (NSA), hip axis length (HAL), and AI.

### Clinical implications

A low LCEA increases fracture risk and necessitates preventive measures such as physiotherapy to enhance hip stability, regular monitoring, or surgical interventions like periacetabular osteotomy (PAO) to improve acetabular coverage. A low FHEI, associated with lateral and pertrochanteric fractures, can be addressed through preventive exercises to centre the femoral head or surgical optimisation of joint biomechanics. An increased femoral canal width, linked to pertrochanteric fractures, requires targeted physiotherapeutic prevention to improve hip stability; in severe cases, prophylactic surgery may be considered. Additionally, elevated BMI should be considered as a risk factor for subtrochanteric fractures, emphasising the importance of weight management in fracture prevention strategies.

### Study limitations

The retrospective nature of this study, its small sample size, and variable image quality may limit the generalisability of the findings. In particular, the absence of a non-fracture control group restricts the applicability of our results to the general population and precludes a direct comparison of anatomical variations that could establish normative values. Furthermore, we did not evaluate specific activity levels of fall mechanisms, as these details were not consistently recorded in the patient’s medical charts. The inclusion of 100 fractures per type may not fully represent the broader patient population. Additionally, osteoporosis diagnoses in our cohort were based solely on available clinical documentation and ICD coding without systematic BMD measurements (e.g., DXA scans), which likely led to an underestimation of osteoporosis prevalence. Prospective studies with larger cohorts are needed to validate these findings and to investigate how different fall mechanisms and patient activity profiles influence proximal femur fracture localisation.

## Conclusion

The study identified key variables associated with proximal femur fracture localisation. A greater HRV increases the risk for lateral fractures, while a lower FHEI increases the risk for lateral, pertrochanteric, and subtrochanteric fractures. An increased femoral canal width, as defined by Dorr, is associated with a higher risk for pertrochanteric fractures. A smaller LCEA also correlates with a higher risk for lateral and pertrochanteric fractures. Importantly, a higher BMI was identified as a significant risk factor for subtrochanteric fractures.
